# Supporting patients receiving oral systemic anti-cancer therapies

**DOI:** 10.1371/journal.pmed.1005022

**Published:** 2026-04-01

**Authors:** Amanda Drury, Matthew Fowler, Janice Richmond

**Affiliations:** 1 Trinity Centre for Practice & Healthcare Innovation, School of Nursing and Midwifery, Trinity College Dublin, Ireland; 2 School of Nursing, Psychotherapy & Community Health, Dublin City University, Ireland; 3 Department of Cancer Services, University Hospitals of Derby and Burton NHS Foundation Trust, Derby, United Kingdom; 4 Department of Oncology, Letterkenny University Hospital, Letterkenny, Co Donegal, Ireland

## Abstract

In this Perspective, Amanda Drury and colleagues outline how and why education, communication, and models of care for patients receiving oral systemic anti-cancer therapies must adapt to support safe, equitable, and sustainable treatment.

## Teaser

Oral systemic anti-cancer therapies improve convenience, but also shift responsibility for treatment management to the patient. Education, communication, and models of care must adapt to support safe, equitable, and sustainable oral anti-cancer treatment.

Traditional systemic anticancer treatments, including intravenous chemotherapy and radiotherapy, often require frequent and prolonged hospital attendance, creating practical burdens for patients and capacity pressures for services. Against this backdrop, oral systemic anti-cancer agents/therapies (oral SACTs) have expanded and are now a central component of cancer treatment across many tumor types. Their increasing use reflects advances in drug development alongside service-level pressures to reduce hospital-based treatment and increase capacity [1].

From a patient perspective, oral therapies are frequently associated with greater convenience, fewer clinic visits, and increased autonomy [2,3]. However, oral SACT also alters how cancer treatment is delivered and managed. Responsibilities that were previously embedded within hospital systems are transferred to patients and their families [3,4]. This shift introduces safety, equity, and quality challenges that are not always visible within routine care pathways [5,6]. A primary safety concern relates to adverse effects and medication problems, including drug-drug interactions with other prescribed medicines, over-the-counter medication, and complementary or herbal products, alongside treatment-taking behavior and persistence [4,5]. Reduced face-to-face contact can make it more difficult for clinical teams to monitor toxicity and to appreciate the complexity of the pill schedules related to cancer and co-morbid conditions, and the practical demands that patients are managing at home [3,4,7]. In this Perspective, we examine how oral SACT can be better supported in practice, arguing that autonomy must be accompanied by structured, ongoing support rather than assumed to be self-sustaining.

The benefits of oral SACT for patients are well recognized, including reduced travel, shorter waiting times, and the ability to integrate treatment into daily routines [2,3]. For many, oral SACT offers improved convenience in treatment and reduces time poverty associated with hospital visits. However, oral treatment relocates a substantial responsibility to patients, who must manage complex regimens; navigate potential drug-drug and drug-food interactions, including interactions with over-the-counter medicines and complementary products; monitor side effects, and judge when symptoms warrant clinical review [4,5]. Studies consistently demonstrate wide variation in continuation and persistence with oral SACT, influenced by symptom burden, toxicity, fatigue, emotional distress, and contextual factors [6,8]. Qualitative research highlights that patients may experience increased uncertainty and isolation, with fewer opportunities to seek reassurance compared with hospital-based care [2,3]. These findings suggest that while oral SACT can enhance autonomy, it simultaneously increases the cognitive and emotional demands placed on patients, particularly in the context of treatment-related symptoms, including fatigue, cognitive impairment, and psychological distress. Without appropriate support, the anticipated benefits of convenience may be offset by this unrecognized burden.

Variation in the use of oral SACT is commonly framed in terms of adherence. However, adherence-based language carries implicit moral judgements, positioning deviation as patient failure, rather than as a response to treatment burden or contextual challenges [6]. This framing may discourage people receiving oral SACT from disclosing missed or delayed doses, particularly when such discussions are not routinely or explicitly invited by healthcare professionals [4]. Clinical assessments undertaken on a recurring basis may over- or under-estimate adherence to oral SACT, particularly when the reasons for missed or delayed doses are not fully explored [4,6]. Where these reviews are conducted by telephone or video, this can further constrain opportunities to assess how people are managing treatment in their own environment and to detect emerging problems early [1,3,7]. However, deviations from prescribed regimens are often a response to unmanaged side-effects, particularly reduced cognition, forgetfulness, competing life demands, or uncertainty about how to manage symptoms [6,8]. Clinical teams should therefore reconceptualize variation in treatment compliance as a potential indicator of unmet need, shifting the focus from monitoring adherence to understanding the persons’ context [6]. Open questioning that explores side-effects, daily routines, and their influence on treatment may enhance communication and trust, and support person-centered care, ensuring treatment is feasible and sustainable within everyday life [3,4,9].

Education is central to safe oral SACT use, yet personalized education is often inconsistent. Essential supportive care information, including guidance on managing missed or vomited doses, recognizing serious adverse effects, and when to seek help, are frequently omitted in routine practice [4]. People living with cancer value education that is practical, empathetic, and responsive to their circumstances, including health literacy, language, and emotional readiness [2,3]. Education and information exchange should be framed as an ongoing process rather than a single event at treatment initiation, with evidence suggesting that education is most effective when combined with follow-up and reinforcement over time [10]. Structured approaches such as teach-back and written care plans support understanding and retention [4]. Education for oral SACT must therefore move beyond information provision towards a structured, person-centered, iterative process that is reinforced to support safe and sustainable treatment use.

Digital interventions, including text reminders, smartphone applications, and web-based platforms, are increasingly promoted to support oral SACT use. Digital tools can be beneficial for some people, with heterogeneous effects across cancer population subgroups [10,11]. However, digital-first approaches risk exacerbating inequities where access, literacy, or trust in technology is limited, determining whether technology supports or hinders self-management [8,10,11]. Low-technology options, such as pill organizers/dosette boxes, paper calendars, and written symptom plans, can support treatment-taking for some people when embedded within supportive care relationships [1,4,10]. However, not all oral SACT can be safely repackaged, and pill organizers may be inappropriate when handling precautions or cognitive impairment are present; in such cases, targeted education for patients and caregivers and nursing oversight are central to safe use [4,7]. Decisions about repackaging, handling precautions, and drug interactions will benefit from coordinated input from oncology pharmacists, nursing teams, and community pharmacists, particularly for patients with polypharmacy or cognitive impairment [4,5]. Digital tools should therefore be understood as adjuncts to high-quality care delivered by skilled healthcare professionals, rather than as substitutes for clinical expertise, relational support, and ongoing professional oversight [5,10,11]. In many settings, the cost of oral SACT and associated care may also translate into substantial out-of-pocket expenses, adding financial burden to the practical and emotional workload of treatment [1,7,8]. While no single model will be appropriate in all contexts, equity and safety for oral SACT care requires adaptable systems that recognize variation in patient capacity and resources.

Evidence increasingly points to the importance of structured, ongoing, multidisciplinary support for patients receiving oral SACT that spans hospital and community services. For example, the AMBORA trial demonstrated that incorporating scheduled counseling sessions with clinical pharmacologists and clinical pharmacists focused on medication management, patient training, side-effect prevention and management, and adherence counseling reduced medication-related problems during treatment with new oral anti-cancer agents [5]. In practice, this multidisciplinary approach requires coordination with community-based clinicians, including general practitioners and community pharmacists, who frequently manage comorbid medications and are well-positioned to identify interaction risks arising from new prescriptions, over-the-counter products, or complementary therapies [4,5]. Nurse-led multidisciplinary models provide further insight into how continuous support can be delivered across settings. A pilot study of a community-based advanced nurse practitioner-led multidisciplinary model of care for adults receiving oral SACT demonstrated high patient and staff acceptability, maintained safety processes, and the feasibility of frequent follow-up outside hospital settings while maintaining links with specialist oncology services [1]. Complementary qualitative studies highlight that continuity, accessibility, and integration across care settings are highly valued by patients and clinicians [7,9]. These findings emphasize the function of follow-up in providing supportive care, supporting early identification of problems, adapting care plans, and maintaining continuity between hospital and community services.

Oral SACTs have transformed how cancer treatment is delivered, offering greater convenience and autonomy for many patients. However, this shift also relocates substantial clinical and cognitive responsibilities from healthcare systems to individuals and families. The benefits of oral SACT are unlikely to be realized through access to medication alone, nor through isolated educational or technological interventions. Instead, safe and sustainable oral SACT use depends on models of care that recognize variation in patients’ capacity, context, and support needs over time. Education should be iterative and reinforced, communication should invite openness rather than compliance, and digital tools should complement, not replace, expert clinical judgement and relational care. As oral SACT use increases, attention must shift from assumptions of self-management towards care systems that actively support patients to use these therapies safely, equitably, and in ways that are feasible within everyday life.

## Abbreviation:

SACT: systemic anti-cancer agent/therapy
